# Icodextrin reduces adhesion formation following gynecological surgery in rabbits

**Published:** 2011

**Authors:** Behnaz Khani, Nahid Bahrami, Ferdous Mehrabian, Hormoz Naderi Naeni

**Affiliations:** 1 Department of Obstetrics and Gynecology, Shahid Beheshti Hospital, Isfahan University of Medical Sciences, Isfahan, Iran.; 2 Department of Obstetrics and Gynecology, Alzahra Hospital, Isfahan University of Medical Sciences, Isfahan, Iran.; 3 Sepahan Hospital, Isfahan, Iran.

**Keywords:** *Icodextrin solution*, *Human amniotic fluid*, *Adhesion formation*, *Rabbit*

## Abstract

**Background:**

Adhesion is a common complication of gynecology surgery so different barrier agents and solutions have been used during these operations to separate and protect tissues from adhesion after surgery. Adept is one of these solutions that have been postulated to reduce the chance of adhesion following gynecolgy surgery.

**Objective:**

To evaluate the effect of 4% icodextrin in reducing adhesion formation in comparing with sterile water and human amniotic fluid in rabbits.

**Materials and Methods:**

In this prospective experimental study 30 white Newzealand female rabbits were selected and randomized in to three treatment groups. The rabbits were anesthetized and an abdominal incison was made, uterine horns were abrated with gauze until bleeding occurred. Before closing the abdomen, the traumatized area was irrigated either by 30cc of sterile water, 30cc of 4% Adept or 30cc of human amniotic fluid. The solutions were labeled only as solutions A (steriel water), B (icodextrin), or C (human amniotic fluid). On the seventh day after surgery, second laparotomy was performed to determine and compare adhesion formation in rabbits.

**Results:**

There was significant difference between mean score of adhesions in 4% icodextrin group (2.1±0.70) in comparison to sterile water group (10.4±0.60) and amniotic fluid group (8.7±0.84). But the difference between mean score of adhesions in amniotic fluid group in comparison to sterile water group was not significant (8.7±0.84) versus (10.4±0.60).

**Conclusion:**

The use of 4% icodextrin solution was more effective than human amniotic fluid and sterile water in reducing adhesion formation in a gynecological surgery model in rabbits

## Introduction

Adhesions are the most common cause of post operative small bowel obstruction, infertility and visceral pain (1). Pelvic surgery is associated with high rates of pelvic adhesion formation. Careful surgical techniques have been proved to reduce adhesion formation. Application of fine, Non-reactive suture materials and prevention of foreign-body reaction, excision of necrotic tissue and minimizing tissue and organ injury are effective in reducing adhesion formation in a surgical procedure (2). 

Non surgical techniques such as application of local and systemic anti-inflammatory agents and peritoneal instillates have been used in this regard. Anti adhesion barriers such as hyaluronic acid, polyethylene glycol, fibrin glue, hyaluronic acid film, and expanded polytetrafluoroethylene have been shown to reduce the incidence and extent of new and recurrent adhesions in different clinical trials (3, 4).

The use of fluids in the peritoneal cavity to separate surfaces and prevent adhesion formation between organs is under investigation. One of these fluids is human amniotic fluid which is a hypotonic solution mainly contains albumin, cholesterol and hyaluoronic acid, existance of hyaluoronic acid in the peritoneal cavity shifts the repair process into regeneration pathway and decreases fibrosis and scar formation. Amniotic fluid also contains some potent growth factors such as insulin like growth factors that are involved in repair process (5). 

In our study, human amniotic fluid was selected and compared with adept adhesion reduction solution that is a pale yellow fluid (icodextrin w/v 4% solution) and is a non viscous, iso-osmotic, clear solution, contains icodextrin, alpha-1, 4 linked glucose polymer with a molecular weight of 16,500 Daltons. 

This product is not physiologically present in the abdominal cavity; it remains in the peritoneal cavity for 3- 5 days before absorption by the lymphatic system and therefore resides longer compared to other solutions such as saline and a glucose-based peritoneal dialysis solution. The existence of 4% icodextrin in the peritoneal cavity during this critical period separates damaged surfaces and minimizes adhesion formation between organs. It gradually absorbs into the blood stream and is broken down by amylase and metabolized to glucose (6).

In a controlled pilot study, the safety and efficacy of 4% icodextrin was evaluated after laparoscopic gynecological surgery and the results showed that it is effective in reducing adhesion formation (7).

Also in another randomized blinded trial anti adhesion efficacy of 4% icodextrin, ferric hyaluronate gel and Ringer lactat were compared in sever peritoneal damage caused by bipolar coagulation in a laparoscopic rat model. Adhesins were more filmier and easily separable in 4% icodextrin group comparing with Ringer lactate group (8).

In contrast to these research results some other working groups found insufficient effects of 4% icodextrin in animal models (9, 10). Two cases of severe serosal fibrosis within a few days after usig 4% icodextrin for reducing adhesion in abdominal surgery was reported (11). Numerous cases of abdominal pain and sterile chemical peritonitis have been contributed to 4% icodextrin (12).

Because the bio compatibility and efficacy of 4% icodextrin is the subject of controversial discussion in the current literature, we planned our study and used a rabbit model to evaluate the effectiveness of 4% icodextrin in reducing adhesion formation in comparing with sterile water and human amniotic fluid.

## Materials and methods

This prospective experimental study was done in Physiology Department of Isfahan University of Medical Sciences, Isfahan, Iran and approved by institutional review board and vice chancellery research of this university by registry number of 386161.

30 white, Newzealand female rabbits, weighing 2000-2200g were randomly assigned to 3 groups. Each group consisted ten non pregnant, 12 weeks aged rabbits. They were fed with standard laboratory rabbit food and water throughout the study. Human amniotic fluid was taken in a sterile condition during cesarean section of two pregnant women who both were around 32 weeks pregnant and had the same indication of cesarean section. To remove red blood cells from the fluid it was centrifuged for ten minutes (3000circules/min) and kept in refrigerator for four hours before use. The 4% icodexterin solution was hydrochloride (Baxter healthcare corporation, Deerfield IL, USA) and the sterile water was from Daroupakhsh Company, Tehran, Iran. The gauze was from Safa Company, Isfahan, Iran.

The rabbits were anesthetized for surgery with IM injection of 55 mg/kg of ketamine. The abdominal ventral side was shaved and dis-infected with povidone iodine. A vertical 5cm abdominal incision was made. Uterine horns were exteriorized and the serosal surfaces of the horns were abraded with sterile gauze until bleeding occured. Up to this point, all animals received the same procedure but after that the injured area were irrigated with different solutions. The solutions were labelled only as A, B or C, so that the study personnel were blinded to solution identity. 

The first group acted as control group in which 30cc of A solution was poured over the traumatized area. In the second group, the damaged area was irrigated with 30cc of B solution and the third group received 30cc of C solution before closure of the abdomen. 

**Measurements**

The second laparotomy was carried out in 30 rabbits after 7 days to assess adhesion formation. The evaluations were blinded for three groups. The formed adhesions were scored by qualitative and quantitative parameters (Table I). Parameters included extent, depth of adhesion, bursting strength, and number of adhesion sites (13). The score from four parameters were calculated and added to define total adhesion score as the grade of adhesion (Table II) (13).

**Statistical analysis**

Adhesion scores were assessed by a blinded surgeon and the mean scores of adhesion were analyzed by SPSS software version 13 using Mann-Whitney test p<0.05 was considered statistically significant.

## Results

In second laparotomy, 7 days later in the sterile water group, the occurrence of severe adhesions was evident. All rabbits in this group showed adhesions, 7 cases (70%) had severe adhesion (grade 3) and 3 cases (30%) had moderate adhesion (grade 2). In 4% icodextrin group, adhesions were found to develop only in 5 rabbits (50%) and half of cases displayed no adhesions at all. Adhesion in these 5 rabbits was merely low grade (grade 1). Finally, in the human amniotic fluid group all rabbits developed some extent of adhesion, 4 rabbits (40%) displayed severe adhesion, and another 4 cases (40%) showed moderate adhesion and the rest, 2 cases (20%) developed mild adhesion. The score of adhesion was calculated for each rabbit as mentioned above. Then the mean score for each group was measured and compared as shown in table III. The mean score of adhesion was (2.1±0.70) for 4% icodextrin group while the mean score was (10.4±0.60) for sterile water group, so the difference was statistically significant (p=0.000) (Table III). 

In human amniotic fluid group the mean score was (8.7±0.84) and in comparison with sterile water group (10.4±0.60), the difference was not significant (p=0.10) (Table III).

 Finally, there was a significant difference between the mean score of adhesion in 4% icodextrin group in comparison to amniotic fluid group.

**Table I:** Qualitative and quantitative measurement

** Score of adhesion**	**Score** **0**	**Score** **1**	**Score** **2**	**Score** **3**
**Adhesion type**				
Extent (mm)	0	<2	2-10	>10
Depth (mm)	0	<1	1-3	>3
Bursting strength	0	+	++	+++
Number of adhesion sites	0	1-2	3-4	>4

**Table II:** Total scoring of adhesion

**Score**	**Adhesion**
4-5	Mild (grade 1)
6-8	Moderate (grade 2)
9-12	Severe (grade 3)

**Table III:** Comparison of adhesion scores among groups

**Groups**	**No. of rabbits**	**Mean ± SD**	**p-value** **vs. sterile water**
Sterile water	10	10.4 ± 0.60	
Adept	10	2.1 ± 0.70	p=0.000
Amnion fluid	10	8.7 ± 0.84	p=0.01

**Figure 1:**
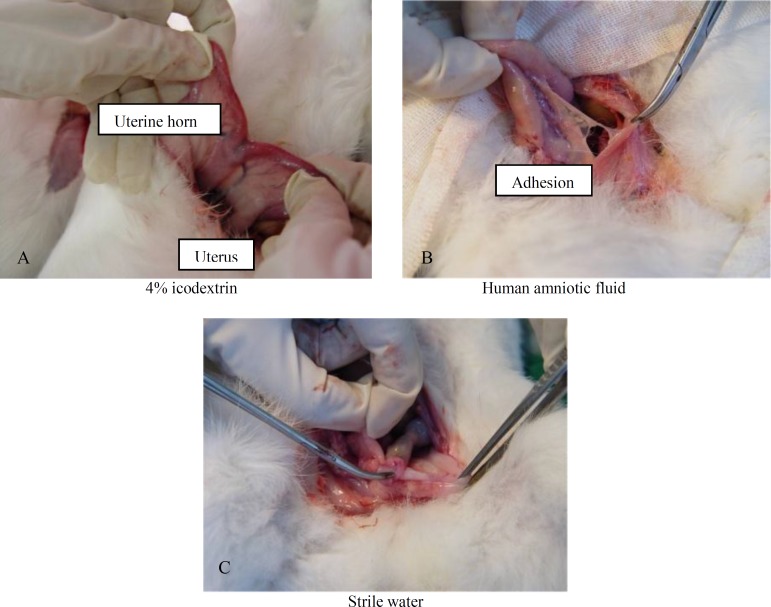
Gross view of adhesion with different treatments

## Discussion

Because adhesion adveresely affects patient morbidity and is a great burden to health system, different techniques have been proposed and tested to reduce adhesion formation (14). Fine surgical techniques and use of laparoscopic surgery to minimize tissue damage are to some extent effective in this regard but Surgical and Clinical Adhesions Research study data showed that this is not sufficient to prevent adhesion formation (15). Administration of specific fluids such as lactated Ringer’s Solution (LRS), phosphate-buffered saline (PBS) and normal saline in to the peritoneal cavity during the surgery has been proposed to reduce formation of adhesions. However these solutions are absorbed in a short period of time and therefore are not effective clinically in preventing adhesion formation (16).

It was shown that administration of human amniotic fluid in to the peritoneal cavity inhibits production of expanded peritonitis. Human amniotic fluid contains hyaluoronic acid that promotes normal healing process (17) and also contains hyaluoronic acid stimulating activator (HASA) which stimulates scar cells to produce hyaluoronic acid that inhibits migration of lymphocytes and prevents chemotaxis and phagocytosis of granulocytes and therefore inhibits scar formation (18).

Four studies commented the prevalence of adhesions at second look laparoscopy (19-22) and showed evidence of decreased prevalence of adhesions in patients who were treated with hyaluronic acid compared with those given placebo or no treatment. 

Our results also indicate that human amniotic fluid is more effective in reducing adhesion formation in comparison with sterill water as placebo. We used 4% icodextrin fluid because it has a longer residual time in the abdominal cavity in comparison to other solutions (23) and it was compared with human amniotic fluid which contains Hyaluronic acid that has been proposed to reduce adhesion formation too. Rabbits were our experimental model because the fluid dynamics of icodextrin in this species is more closely similar to human beings (24). 

Early pre-clinical studies were performed (by Verco *et al* 2000) to assess the efficacy of 4% icodextrin in order to reduce adhesion in a rabbit double uterine horn model (25). Their results indicate that postoperative application of icodextrin 4% causes a significant increase in adhesion free sites (p=0.000). 

In a randomized controlled study (by Dizerega *et al* 2003) the safety and efficacy of 4% icodextrin was evaluated. In order to compare icodextrin 4% with Ringer’s lactated saline, 62 women who required laparascopic adnexal surgery were compared in two different treatment groups. Results showed that lavage and instillation with icodextrin 4% was effective in reducing adhesion formation. The use of 4% icodextrin solution for peri operative lavage and post operative instillation in rabbit model of bowel anastomatic healing, didn’t result in any difference from either LRS treated or untreated surgical control (26).

In another study (by Muller *et al* 2005) the effect of intraperitoneal anti adhesive fluids (4% icodextrin, phospholipids, Ringer’s lactate) in a rat peritonitis model was examined and the results of 4% icodextrin showed significantly enhancement of adhesion and abscess formation, in comparison with the other control groups. A case of disseminated intra vascular coagulation after laparoscopic multiple myomectomy with the use of 4% icodextrin solution was described by Santos *et al* (2006). The possible cause might be idiosyncratic immunologically mediated reaction to icodextrin in the pelvic cavity, but no previous case of DIC has been described in the published litreture (27). Some cases of vulval edema, plural effusion and even anaphylactoeid reaction related to icodextrin 4% after laparoscopic and laparotomy surgery have been reported (28).

But, ARILE (Adept registry for clinical evaluation) was initiated in a number of centers in the UK and then expanded to involve 253 centeres (103 general-surgery and 150 gynecologyical-surgery centers) in France, Germany, Italy, Spain, Greece (gynecology only) and the UK. The findings indicate that icodextrin 4% was well tolerated by patients who underwent laparotomy or laparoscopy (29).

In a recent study (by Colins *et al* 2007), 402 patients randomized intraoperatively to receive either 4% icodextrin or LRS and then patients returned for second laparoscopy within 4–8 weeks. Incidence, severity, and extent of adhesions were characterized for both groups and they demonstrated that 4% icodextrin is a safe and effective adhesion reduction agent in laparoscopy (30).

Our study results support preclinical observation (by Verco *et al* 2000) and recent study (by Colin *et al* 2007), as mentioned before in adept group, half of the cases were found with no adhesion at all and the rest had mild adhesion.

However in our study, no side effects, no abscess formation and no other complication were observed in contrast to Muller *et al* (2005). Our data showed that lavage and instillation of 4% icodextrin was not only safe but also effective in reducing adhesion formation in rabbits, and with regard to close relationship of fluid dynamics in rabbits with human being this results suggest that patients undergoing gynelocological surgery may have a better prognosis for adhesion reduction after using intra operative irrigation with 4% icodextrin.
